# The Eruption of the Permanent Teeth

**Published:** 1886-01

**Authors:** C. R. Butler


### The Eruption of the Permanent Teeth.

Though a marked irregularity in the temporary teeth is unusual, a fairly arranged permanent set among persons of American nationality is quite the exception.

The first molars of the permanent set are usually erupted at the age of six, with crowns larger than those of the teeth cut at a later date, they serve a very important function, their roots being fully formed before the shedding of the temporary teeth, they sustain the work of mastication between childhood and youth, giving the necessary stimulus to the salivary glands, and thereby causing the food to be properly insalivated before deglutition. They also prevent undue pressure upon the teeth of replacement. The jaws having elongated, they are enabled to take up position just behind the twenty milk or temporary teeth.

A further elongation between the ages of six and twelve, makes room for the second and third molars, to which latter, the dentes sapientia, I now draw your attention.

The eruption of the inferior wisdom teeth which are situated in the angle of the inferior maxillary frequently causes much discomfort.

The general structure and arrangement of the full complement of thirty-two teeth is a fair index of the anatomical and physiological make up of the individual.

The shortening and narrowing of the lower portion of the face, and consequent want of room for the teeth in the maxillary bones is apparent to the most casual observer of the present American race, and the ailments that confront the general practitioner and specialist are increasingly of a nervous character.

To discover a means of correcting this deformity, and alleviating the consequent disease, is the prime object of our meeting.

A diseased tooth is often the cause of serious trouble, both local and general, through nerve irritation.

The typal number remains unchanged, and the size of the teeth has not decreased along with the diminution of the jaw.

It has been said with reason, that a frequent cause of irregularity and decay of the teeth lies in the inheritance of incongruous jaws and teeth from the parents. The father having large teeth in a large jaw, and the mother small teeth in a small jaw, though both may have perfect sets, the result is most unfortunate for the children, the teeth being as a rule inherited from the father and the jaw from the mother.

In consequence of the limited space between the second molar and the base of the outer or vertical portion of the ramus, the erupting third appearing at the age of eighteen or twenty, or even earlier, are forced against the lateral ends of a fixed circle, causing serious inflammation and the necessity for surgical relief.

The only treatment suggested by the text-books or journals of dental surgery, is the extraction of the second molar.

To sacrifice a tooth or teeth of the most important class to make room for one situated on the end of the jaw with imperfect occlusion, seems, except in extreme cases, an unwise proceeding.

The dento maxillary nerves being distributed from the casserean ganglion it is easy to see how reflex nerve irritations is caused by one of these offending teeth.

In cases that have come under my treatment during the past year, I have observed the following effects: Aggravated throat troubles, deafness, stiffening of the muscles of the face and neck, facial neuralgia, local paralysis, prostrating pain, with severe abscess, and other local and systemic disturbances, caused by one or more of these impacted teeth.

Young women especially suffer at the approach of the menstrual period.

In a case of great suffering from nervous troubles which had not yielded to treatment, there were experienced a dull pain in the back of the head and neck and a difficulty in swallowing. The third molars above and below were removed, and relief speedly followed.

Where the parts are inflamed, it is well to resort to a depletion of the gum by clipping away the overlying portion until extraction can be effected.

The superior dentis sapientia often demand attention. By a want of room they are forced outwards, causing muscular irritation by the excoriation of the inner portion of the cheek, they, however, are more readily removed than those of the lower jaw.

---

On motion of Dr. J. Taft, President James appointed Drs. Butler and Taft Committee on Installation, to conduct the President elect, Dr. Rehwinkel to the chair, the latter making some appropriate remarks, as also did Secretary Callahan.

The President, Dr. Rehwinkel, then announced the following Standing Committees:

*Arrangements.*

Harroun, Toledo.
A. Terry, Norwalk.
J. E. Robinson, Cleveland.

*Public and Voluntary Essays.*

J. Taft.
E. G. Betty.
J. R. Callahan.

*Membership.*

C. R. Butler.
C. H. James.
F. C. Runyan.

On resolution of J. Taft, it was decided to hold the next annual meeting at Toledo—last Tuesday in October, 1886.

Adjourned.

